# Functional properties of measles virus proteins derived from a subacute sclerosing panencephalitis patient who received repeated remdesivir treatments

**DOI:** 10.1128/jvi.01874-23

**Published:** 2024-02-08

**Authors:** Katharina S. Schmitz, Kim Handrejk, Lelde Liepina, Lisa Bauer, Griffin D. Haas, Fabiënne van Puijfelik, Edwin J. B. Veldhuis Kroeze, Marta Riekstina, Jurgis Strautmanis, Huyen Cao, Robert M. Verdijk, Corine H. GeurtsvanKessel, Sander van Boheemen, Debby van Riel, Benhur Lee, Matteo Porotto, Rik L. de Swart, Rory D. de Vries

**Affiliations:** 1 Department of Viroscience, Erasmus MC, Rotterdam, The Netherlands; 2 Clinic for Pediatric Neurology and Neurosurgery, Children’s Clinical University Hospital, Riga, Latvia; 3 Department of Microbiology, Icahn School of Medicine at Mount Sinai, New York, New York, USA; 4 Department of Pathology, Children’s Clinical University Hospital, Riga, Latvia; 5 Departments of Clinical Research, Biometrics, and Virology, Gilead Sciences, Inc., Foster City, California, USA; 6 Department of Pathology, Erasmus MC University Medical Center Rotterdam, Rotterdam, The Netherlands; 7 Department of Pediatrics, Columbia University Irving Medical Center, New York, New York, USA; 8 Center for Host–Pathogen Interaction, Columbia University Irving Medical Center, New York, New York, USA; 9 Department of Experimental Medicine, University of Campania “Luigi Vanvitelli”, Caserta, Italy; University of Kentucky College of Medicine, Lexington, Kentucky, USA

**Keywords:** measles, subacute sclerosing panencephalitis, remdesivir, fusion, resistance

## Abstract

Subacute sclerosing panencephalitis (SSPE) is a rare but fatal late neurological complication of measles, caused by persistent measles virus (MeV) infection of the central nervous system. There are no drugs approved for the treatment of SSPE. Here, we followed the clinical progression of a 5-year-old SSPE patient after treatment with the nucleoside analog remdesivir, conducted a post-mortem evaluation of the patient’s brain, and characterized the MeV detected in the brain. The quality of life of the patient transiently improved after the first two courses of remdesivir, but a third course had no further clinical effect, and the patient eventually succumbed to his condition. Post-mortem evaluation of the brain displayed histopathological changes including loss of neurons and demyelination paired with abundant presence of MeV RNA-positive cells throughout the brain. Next-generation sequencing of RNA isolated from the brain revealed a complete MeV genome with mutations that are typically detected in SSPE, characterized by a hypermutated M gene. Additional mutations were detected in the polymerase (L) gene, which were not associated with resistance to remdesivir. Functional characterization showed that mutations in the F gene led to a hyperfusogenic phenotype predominantly mediated by N465I. Additionally, recombinant wild-type-based MeV with the SSPE-F gene or the F gene with the N465I mutation was no longer lymphotropic but instead efficiently disseminated in neural cultures. Altogether, this case encourages further investigation of remdesivir as a potential treatment of SSPE and highlights the necessity to functionally understand SSPE-causing MeV.

**IMPORTANCE:**

Measles virus (MeV) causes acute, systemic disease and remains an important cause of morbidity and mortality in humans. Despite the lack of known entry receptors in the brain, MeV can persistently infect the brain causing the rare but fatal neurological disorder subacute sclerosing panencephalitis (SSPE). SSPE-causing MeVs are characterized by a hypermutated genome and a hyperfusogenic F protein that facilitates the rapid spread of MeV throughout the brain. No treatment against SSPE is available, but the nucleoside analog remdesivir was recently demonstrated to be effective against MeV *in vitro*. We show that treatment of an SSPE patient with remdesivir led to transient clinical improvement and did not induce viral escape mutants, encouraging the future use of remdesivir in SSPE patients. Functional characterization of the viral proteins sheds light on the shared properties of SSPE-causing MeVs and further contributes to understanding how those viruses cause disease.

## INTRODUCTION

Measles, caused by measles virus (MeV), a member of the family *Paramyxoviridae*, is a highly contagious acute disease characterized by respiratory symptoms, fever, rash, and immunosuppression. The acute phase pathogenesis of measles is primarily defined by the use of two cellular entry receptors: signaling lymphocyte activation marker F1 (SLAMF1, CD150) and nectin-4, which are expressed on myeloid, lymphoid, and epithelial cells (1–3). Because of the preferential infection and depletion of memory lymphocytes by MeV, followed by a phase of “immune amnesia”, measles morbidity and mortality are mainly caused by bacterial infections after measles. In rare cases, MeV spreads to the central nervous system (CNS), despite the lack of CD150 and nectin-4 expression, leading to neurological complications that can occur acutely, weeks to months or only years after initial MeV infection (4, 5).

A rare but fatal neurological complication of measles is subacute sclerosing panencephalitis (SSPE), which typically develops 4–10 years after MeV infection. Its average incidence is between 1:4,000 measles cases, which increases to 1:270 in children who contracted measles under the age of 1 year (6–9). SSPE is always fatal; less than 50% of patients diagnosed with SSPE survive 2 years post-diagnosis and less than 20% more than 4 years (10). SSPE can be divided into four progressive clinical stages. Stage 1 is characterized by personality changes, mood swings, or depression, followed by myoclonic seizures and spasms (Stage 2). In Stage 3, seizures are replaced by twisting movements and rigidity, and in Stage 4, the patient’s brain is progressively damaged leading to coma and eventually death. Patients develop hyper-immune responses to MeV, including intrathecal production of MeV-specific antibodies, frequently used as diagnostic criteria (11–13).

There is no licensed treatment for SSPE. Experimental treatment of SSPE patients with compounds including antiviral and immunostimulatory drugs, like inosine pranobex, type I interferons, ribavirin, and other nucleic acid analogs, has largely been ineffective (14). The nucleoside analog prodrug remdesivir, which was also used during the SARS-CoV-2 pandemic to treat patients with moderate and severe COVID-19 (15), has antiviral activity against MeV *in vitro* and could potentially be used in the treatment of SSPE (14, 16, 17). However, prevention of primary MeV infection by vaccination is the best option to avert SSPE; safe and effective live-attenuated vaccines are available and have never been demonstrated to cause SSPE. Yet, relying on vaccination for SSPE prevention is hampered by two challenges. First, vaccination coverage gaps have led to a resurgence of measles. During the COVID-19 pandemic, cases dropped significantly due to non-pharmaceutical intervention methods, but as several vaccination campaigns were suspended, millions of children are now at risk of developing measles and, thus, SSPE in the post-COVID-19 era (18, 19). Second, the live-attenuated measles vaccine is not effective when administered in the presence of maternal antibodies. These antibodies usually wane around 6 months after birth, and the vaccine is often administered (more than) 12 months after birth. An immunity gap remains, during which unvaccinated infants are at risk of contracting measles early in life (20).

MeV has a single-stranded negative-sense RNA genome encoding six structural and two non-structural proteins (21). MeV has two envelope transmembrane glycoproteins, hemagglutinin (H) and fusion (F). H binds the cellular receptors; F is produced in a metastable state and catalyzes the merging of the viral envelope with the target cell membrane. Together, H and F form the fusion complex that enables viral entry into host cells. The nucleo- (N), phospho- (P), and large (L) proteins make up the ribonucleoprotein complex necessary for the protection of the genomic RNA and its replication and transcription. The M protein orchestrates the assembly of viral particles, initiating the budding of virions at the cell membrane. The two non-structural proteins V and C, transcribed from the P gene, are less well characterized but likely function as virulence factors in the host cell.

SSPE-causing MeV (SSPE viruses) harbor various mutations in their genome. Mutations in the F gene render the protein to be hyperfusogenic, enabling fusion in the absence of known MeV receptors and allowing progressive cell-to-cell spread of MeV genomes throughout the CNS (22–25). Hyperfusogenicity has been associated with decreased stability of the F protein (23, 26–28); the conformational change from the pre- to post-fusion state is triggered more quickly in these F proteins compared to “normal” F proteins. In this context, substitutions that render the F protein hyperfusogenic also destabilize it. Additionally, hypermutation of the M gene is characteristic for SSPE. This leads to the ablation of the production of cell-free viruses, leaving cell-to-cell spread as the only option for the virus to disseminate throughout the CNS (29, 30). Those mutations are often induced by the immunomodulatory host enzyme adenosine deaminase acting on RNA (ADAR) (31).

Here, we describe the case of a 5.5-year-old boy who was diagnosed with SSPE and treated with remdesivir on a compassionate use basis. The treatment transiently improved the clinical course of the disease, but the patient ultimately succumbed to his infection. We performed an in-depth post-mortem histopathological examination of the brain, combined with a functional analysis of the mutated MeV F and L genes to characterize the causative agent and contribute to a better understanding of SSPE viruses.

## RESULTS

### Transient clinical improvement after remdesivir treatment

**Fig 1:**
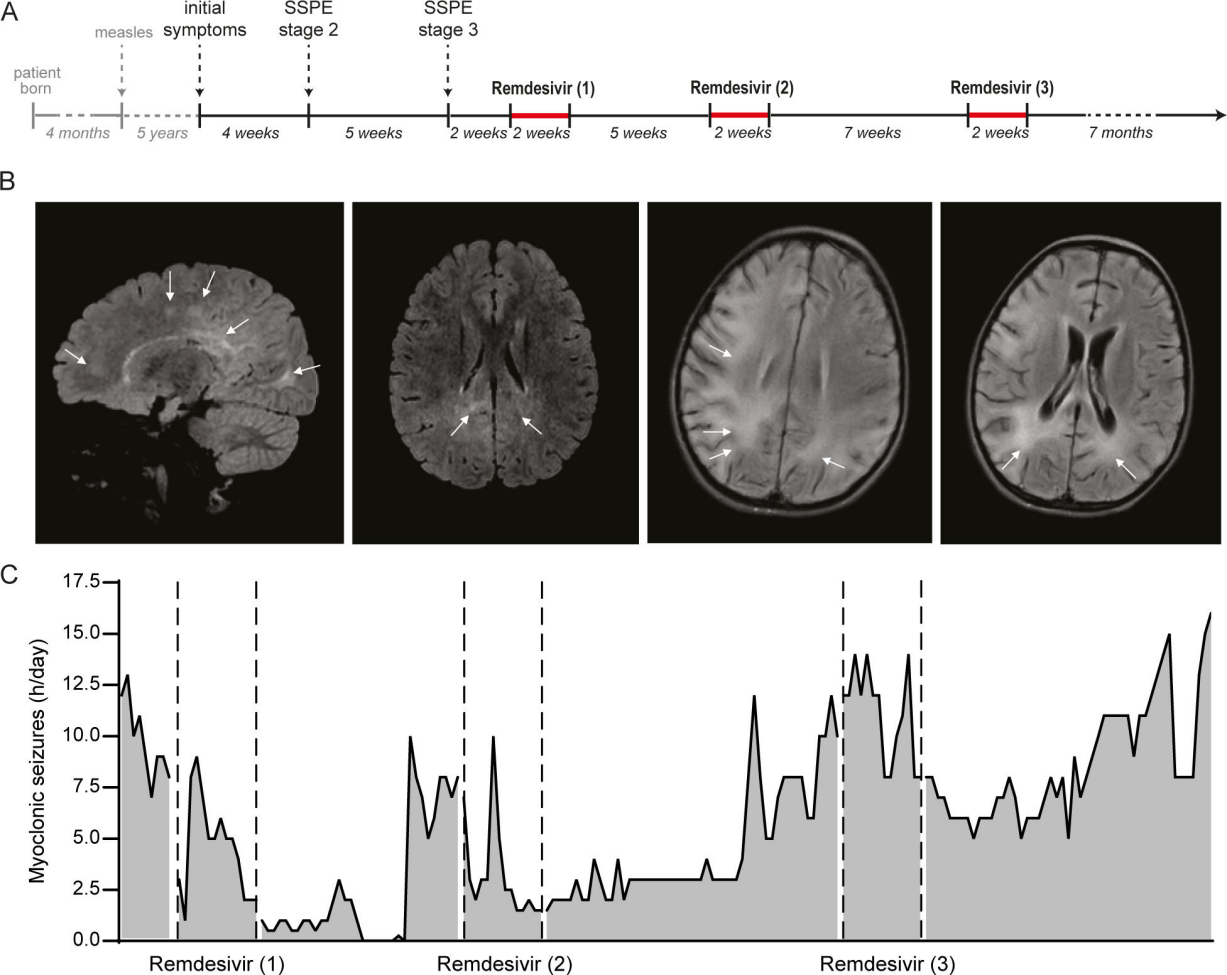
Treatment regimen and clinical parameters. (**A**) Timeline of disease and treatment regimen. (**B**) Attenuated inversion recovery MR images taken upon patient admission (two left panels) and at the time of treatment onset (two right panels). The interval between both scans is 2 months. Two left panels (arrows): diffuse deep periventricular white matter hyperintensities in parietal lobes; bilaterally, subcortical white matter hyperintensities in frontal lobes and white matter hyperintensities involving dorsomedial thalamus bilaterally. Two right panels (arrows): progressing white matter hyperintensities, slight cerebral and cerebellar atrophy, and enlarged lateral ventricles. (**C**) Myoclonic seizures as a measurement for treatment success. Seizures transiently decreased after treatment courses 1 and 2.

A 5.5-year-old Latvian boy without comorbidities, who experienced uncomplicated measles at the age of 4 months, presented with progressive behavioral changes, aggressiveness, regression in scholastic performance, ataxia, and myoclonic seizures. His electroencephalogram revealed characteristic periodic, stereotyped high-voltage discharges, delta complexes (*Rademecker complexes*), and diffuse slowing. Fluid-attenuated inversion recovery magnetic resonance imaging (MRI) showed diffuse deep periventricular white matter hyperintensities in parietal lobes bilaterally, subcortical white matter hyperintensities in frontal lobes, and white matter hyperintensities involving the dorsomedial thalamus bilaterally. Cerebrospinal fluid (CSF) protein levels were normal (0.18 g/L), the MeV genome was detected by reverse-transcriptase polymerase chain reaction (RT-PCR) on CSF (Ct 33.9), and high levels of MeV-specific antibodies were detected in both CSF and serum (Fig. S1). Finally, a CSF-serum antibody index above 3 (4.99) was indicative for intrathecal IgG antibody production against MeV (13). Consequently, the patient was diagnosed with Stage 2 SSPE (11) and treated according to the standard of care with isoprinosine, subcutaneous interferon alpha-2 (IFN2α), and carbamazepine. Within 2 months after presentation, the patient progressed to Stage 3 SSPE (Fig. 1A). He developed myoclonic status epilepticus, experienced increasing rigidity and spasticity in all extremities, and became unresponsive to his surroundings. An MRI revealed progressing white matter hyperintensities, slight cerebral and cerebellar atrophy, and enlarged lateral ventricles (Fig. 1B).

The patient received three courses (each 14 days) of remdesivir on a compassionate use basis, leading to transient clinical improvement after the first two treatments (Fig. 1C). Improvement was most prominent after the first course, myoclonic seizures and tonic nocturnal seizures completely disappeared. The previously non-responsive patient consciously started to speak simple words, understood easy instructions, smiled, and could move his legs and arms voluntarily again despite severe ataxia and dysmetria. Clinical improvement after the third course of treatment was absent. At this point, the patient developed multiple gastrointestinal co-infections and pneumonia leading to further deterioration. The patient died 14 months after onset of symptoms.

### SSPE-related histopathological changes in the brain

**Fig 2:**
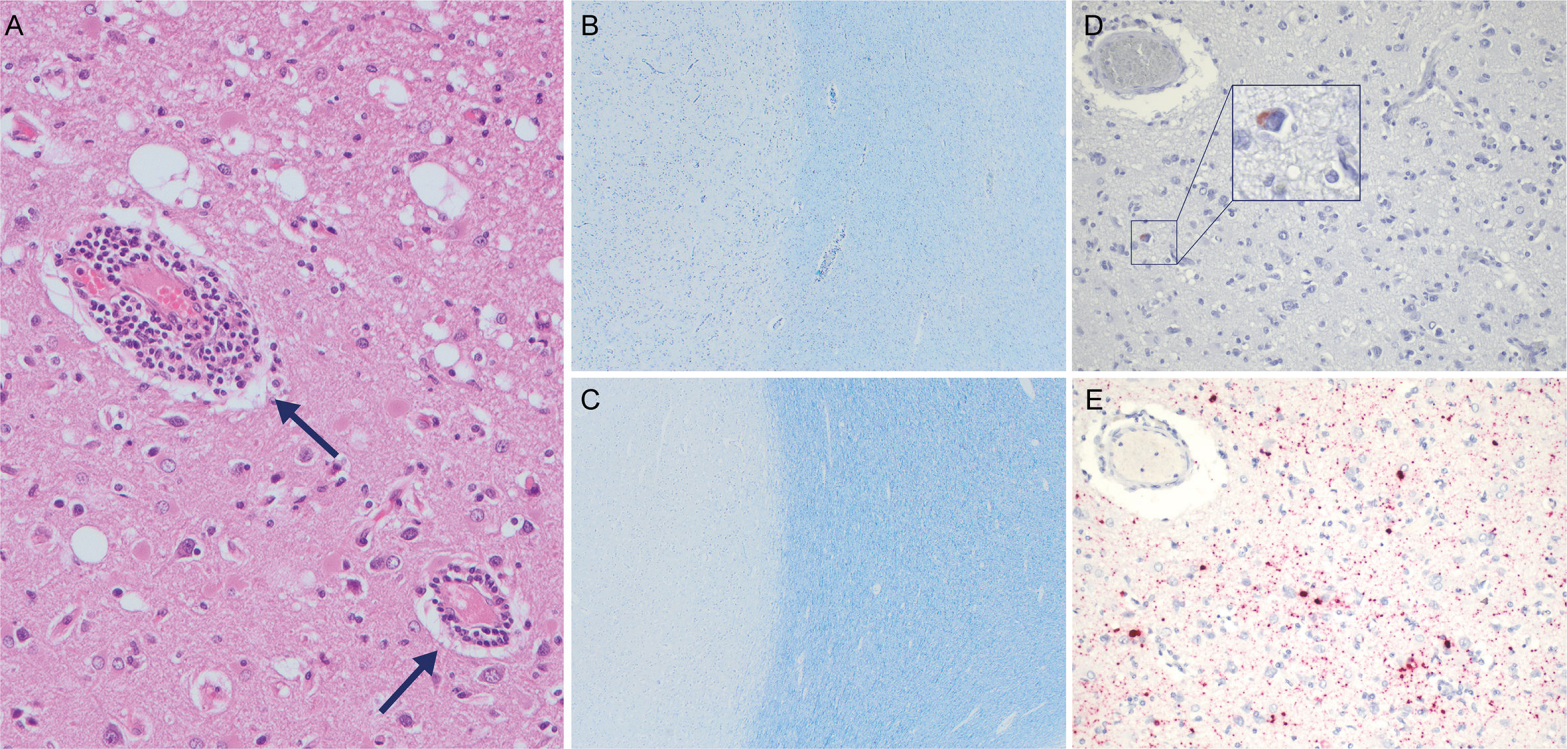
Histopathological evaluation of the brain. (**A**) Hematoxylin and eosin staining of the cerebral parietal lobe revealed multifocal lymphoplasmacytic perivascular cuffing (arrow). (**B and C**) Kluver-Barrera staining to visualize myelin for patient (**B**) and age-matched control (**C**). Gray matter is shown by light staining on the left and white matter by darker staining on the right. Both sections originate from the same anatomical location, the occipital lobe. (**D and E**) Anti-MeV nucleoprotein detection in consecutive slides of the temporal lobe shown by (D) immunohistochemistry (protein staining) or (E) RNA *in situ* hybridization (RNAscope).

A thorough post-mortem examination of the brain (parietal lobe, temporal lobe, occipital lobe, frontal lobe, basal ganglia, cerebellum, hippocampus, corpus callosum, brain stem, and periventricular regions) was performed. Histopathological evaluation showed lymphoplasmacytic perivascular cuffs in the meninges and throughout the brain (Fig. 2A), accompanied by loss of neurons, gliosis, and white matter vacuolation. No evident viral inclusion bodies or multinucleated cells were observed. Interestingly, the myelin staining intensity of the patient’s white matter was less intense when compared to an age-matched control, indicative of demyelination (Fig. 2B and C).

### Abundant detection of MeV RNA in SSPE brain

Next, we analyzed multiple anatomic regions of the brain for the presence of MeV-N protein and RNA. Interestingly, immunohistochemical analysis demonstrated the only sporadic presence of antigen-positive cells in different parts of the brain (Fig. 2D; Fig. S2; Table S1). In contrast, *in situ* RNA hybridization showed abundant presence of MeV nucleoprotein RNA-positive cells especially in the frontal, parietal, and temporal lobes and basal ganglia (Fig. 2E; Fig. S2; Table S1). No protein or RNA was detected in a deep cervical lymph node and the tonsils. A semi-quantitative scoring confirming the presence or absence of antigen-positive and RNA-positive cells in different parts of the brain is shown in Table S1.

### MeV genome contains characteristic SSPE virus features

A complete viral genome was obtained from brain material by Illumina sequencing (GenBank accession number: ON024067). The MeV genome clustered with sequences of genotype B3, which was the dominant circulating MeV strain in Latvia at the time of the primary MeV infection of the patient. The closest related wild-type (WT) MeV strain was MVs/California.USA/05.14/[B3] (accession number: KY969477.1), which served as a reference sequence for further analysis. We observed a total of 158 changes from the reference sequence throughout the coding sequence (Fig. S3; Table S2). A total of 113 changes (71.52%) were detected in the M gene, 13 (8.2%) in the F and L genes, nine (5.7%) in the H gene, and six (3.8%) and four (2.5%) in the N and P genes, respectively. The highest number of changes in relation to the size of the protein was in the M gene, while the lowest was in the L gene. Biased hypermutation dominated by uridine-to-cytosine transitions (suggestive of RNA editing by ADAR) was observed throughout the genome but was especially prominent in the M gene.

### SSPE-L substitutions do not confer resistance to remdesivir

**Fig 3:**
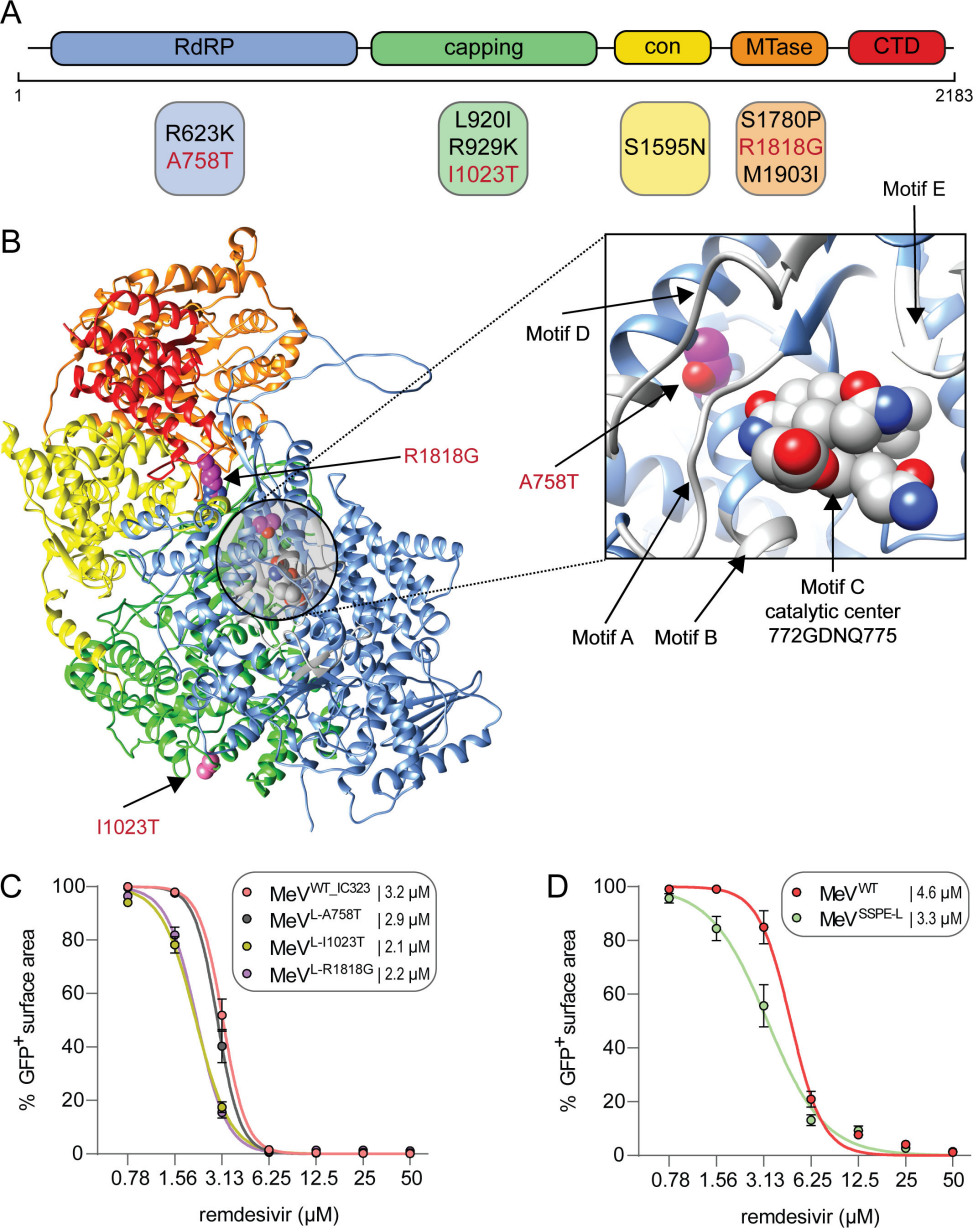
Mapping and evaluation of mutations in the L gene. (**A**) Schematic overview of missense mutations in functional polymerase domains. Mutations shown in red were functionally evaluated. (**B**) AlphaFold-predicted model of the WT MeV RdRP with zoom-in on the catalytic center. The individual domains are colored according to **A**, and the functionally evaluated substitutions are shown in purple. (**C and D**) Remdesivir resistance evaluation of single-point mutation MeVs (**C**) and MeV harboring all major mutations in L (**D**) in comparison to WT MeV. Dose-response curves for remdesivir are shown for an average of *n* = 6 (**C**) and *n* = 9 (**D**) replicates performed in two (**C**) and three (**D**) independent experiments. Error bars depict the SEM. Respective IC_50_ values are presented in the legend.

As the viral polymerase is the target of remdesivir, we next evaluated whether mutations in the L gene conferred remdesivir resistance, potentially responsible for the reduced success of the third treatment course. Nine out of the 13 identified mutations were missense mutations, of which four (A758T, I1023T, S1780P, and R1818G) led to a change in the amino acid properties (charge, hydrophobicity, and polarity) (Fig. 3A). S1780P was not further evaluated since most MeV^WT^ B3 viruses, but not the reference strain, encode a proline on position 1780, suggesting S1780P to be a polymorphism. We performed an AlphaFold prediction to determine the location of the remaining three amino acid substitutions in the context of the predicted polymerase complex. A758T was located in the RNA-dependent RNA polymerase (RdRP) domain and predicted to be in close proximity to the catalytic center. I1023T mapped to the capping domain and R1818G, which was a minority substitution present in 36% of the reads, to the methyl transferase domain (Fig. 3B).

We generated recombinant MeVs (rMeVs) bearing one of these three selected SSPE substitutions in L (rMeV^L-A759T^, rMeV^L-I1023T^, and rMeV^L-R1818G^) and evaluated them for remdesivir resistance compared to rMeV^WT_IC323^ *in vitro*. No difference in remdesivir sensitivity between rMeV^WT_IC323^ and rMeV^L-A759T^ could be detected at 72 hours post-inoculation (hpi) (IC_50_: 3.2 vs 2.9 µM); surprisingly, rMeV^L-I1023T^ and rMeV^L-R1818G^ appeared to be more sensitive to remdesivir compared to rMeV^WT_IC323^ (IC_50_: 2.1 and 2.2 µM) (Fig. 3C). We concluded that there was no indication that the SSPE virus acquired single remdesivir resistance mutations. To exclude a cumulative effect of all SSPE-L mutations, we cloned all eight major coding mutations into a MeV B3 backbone (rMeV^SSPE-L^) and compared its remdesivir sensitivity to a WT B3 MeV (rMeV^WT^). A slightly higher remdesivir sensitivity of rMeV^SSPE-L^ compared to rMeV^WT^ was observed (IC_50_: 3.3 vs 4.6 µM), confirming results obtained with the single mutants (Fig. 3D). Finally, we investigated whether remdesivir resistance mutations could be induced *de novo* by *in vitro* passaging an rMeV based on a clinical isolate from Khartoum Sudan [genotype B3 (32, 33)] in the presence of increasing remdesivir concentrations for more than 20 passages. After passaging, we did not detect the differences between MeV passaged in the presence or absence of remdesivir in an antiviral assay (data not shown). Taken together, our findings suggest that the lower treatment efficacy of the third course of remdesivir was not due to acquired drug resistance.

### SSPE-F substitutions mediate fusion in the absence of known MeV receptors

**Fig 4:**
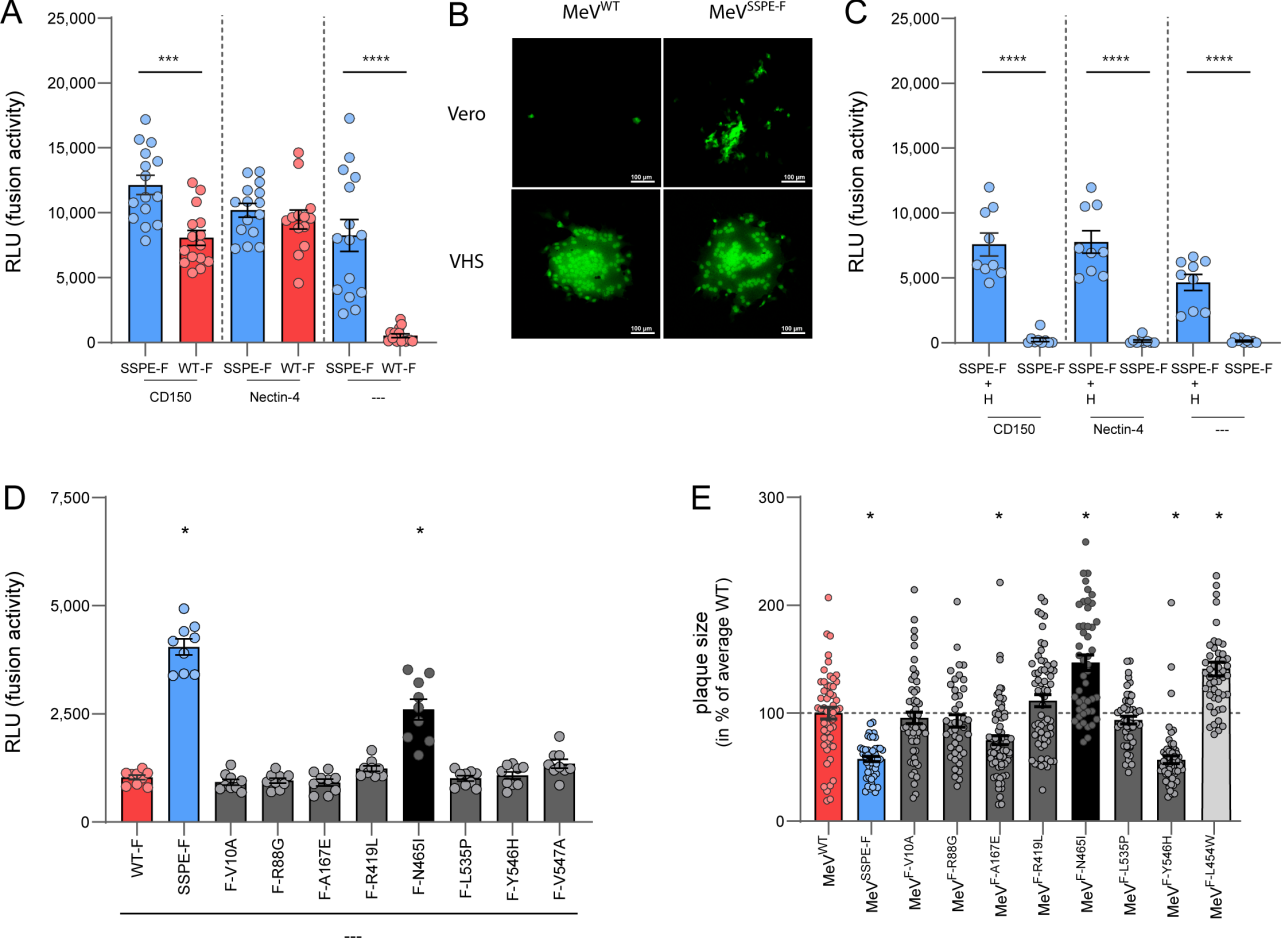
Functional evaluation of mutations in the F gene. (**A**) Complementation-based fusion assays to determine the potential of the MeV fusion machinery to induce cell-to-cell fusion. Cell-to-cell fusion was evaluated for the SSPE-F and WT-F protein co-expressed with a WT-H protein in the presence or absence of CD150 or nectin-4. (**B**) Representative images of syncytium formation after inoculation of Vero (top) or VHS (bottom) cells with rMeV^WT^ (left) and rMeV^SSPE-F^ (right). Syncytia are shown in green. Images were taken 7 dpi (Vero) or 2 dpi (VHS) using a Zeiss LSM 700 confocal microscope at 10× magnification. The bar indicates 100 µm. (**C**) Complementation-based fusion assays to determine the potential of the MeV fusion machinery to induce cell-to-cell fusion in the presence or absence of H. Cell-to-cell fusion was evaluated for the SSPE-F protein co-expressed with or without a WT-H protein in the presence or absence of CD150 or nectin-4. (**A and C**) Unpaired *t*-test. *P ≤ 0.05, **P ≤ 0.01, ***P ≤ 0.001, and ****P ≤ 0.0001. (**D**) Complementation-based fusion assays to determine the potential of the F protein amino acid substitutions to induce cell-to-cell fusion. Cell-to-cell fusion was evaluated for F proteins harboring single amino acid substitutions co-expressed with a WT-H protein in the absence of MeV receptors. WT-F and SSPE-F were used as a control. Unpaired *t*-test compared to WT-F, adjusted *P* ≤ 0.006. (**E**) Relative plaque sizes are shown for rMeV-F single mutants and rMeV^SSPE-F^ compared to rMeV^WT^. Plaques were measured 3 dpi on VHS. Mann-Whitney test compared to WT-F, adjusted *P* ≤ 0.006. (**A, C, D, E**) All experiments were performed in triplicates in at least three independent experiments. Dots depict individual values and error bars the SEM.

Next to mutations in the L gene, several mutations were detected in the F gene. Mutations in the F gene have been described in other SSPE viruses (22, 24, 26, 27, 34–38), which often led to altered fusogenic properties. We performed a complementary fusion assay to characterize these properties of the patient-derived SSPE-F protein co-expressed with a WT-H protein, in combination with the requirement for receptor engagement. The patient SSPE-F-mediated fusion was significantly better or performed similarly when compared to WT-F in the presence of CD150 or nectin-4, respectively (Fig. 4A). In cells lacking expression of the cellular receptors CD150 or nectin-4, SSPE-F was still able to mediate high levels of fusion, whereas WT-F failed to do so. To confirm that the patient SSPE-F could fuse independent of receptor engagement, we generated an rMeV with the SSPE-F protein (rMeV^SSPE-F^). We infected both receptor-negative Vero cells and receptor-positive Vero-human SLAM (VHS) cells with rMeV^WT^ and rMeV^SSPE-F^. As expected, rMeV^WT^ spread rapidly in VHS cells but only sporadically infected single cells in Vero cells and did not spread. rMeV^SSPE-F^ formed similar-sized syncytia in VHS cells compared to rMeV^WT^, and also small syncytia in Vero cells, although the formation of those syncytia took considerably longer (2 vs 7 days) (Fig. 4B).

The H protein is responsible for attaching MeV to the target cell and triggering F to undergo conformational changes and mediate fusion. To assess whether fusion by SSPE-F was dependent on MeV-H, a fusion assay was performed in which SSPE-F was overexpressed in the presence or absence of WT-H. Although the overall luminescence was lower than previously observed, SSPE-F only mediated fusion in the presence of H, independent of the presence or absence of known MeV receptors. This confirmed that this patient SSPE-F was hyperfusogenic and could fuse receptor independently but still required the presence of MeV-H (Fig. 4C).

### The N465I substitution is responsible for the SSPE-F phenotype

Eight major missense mutations were detected in the hyperfusogenic SSPE-F compared to WT-F. We performed the complementary fusion assay described above, overexpressing plasmids encoding a single SSPE-F mutation in the absence of known MeV receptors, to determine whether an individual substitution was responsible for the hyperfusogenic phenotype of the patient SSPE-F (Fig. 4D). Whereas most SSPE-F single amino acid substitutions did not trigger fusion significantly more efficiently compared to WT-F, F^N465I^, which was previously described to lead to a hyperfusogenic phenotype (39), reconstituted more than half of the fusion phenotype.

### rMeV bearing SSPE-F has reduced cell-to-cell spread in CD150-expressing cells

To characterize the influence of the single amino acid substitutions on cell-to-cell spread in the presence of a receptor, we performed a plaque assay in VHS cells. To this end, we generated viruses with single SSPE-F point mutations and compared these to rMeV^WT^ and rMeV^SSPE-F^. rMeV^F-L454W^ was used as a control as it was previously shown to harbor an F protein that is associated with hyperfusogenicity and large syncytium formation (26, 27). Of the single-point mutation viruses, rMeV^F-V547A^ could not be evaluated as no virus stock could be generated. Infection with mutants rMeV^F-Y546H^ and rMeV^F-A167E^ led to significantly smaller plaques compared to rMeV^WT^. In contrast, rMeV^F-N465I^, the virus harboring the substitution previously shown to be responsible for hyperfusogenicity, formed significantly larger plaques than rMeV^WT^ (Fig. 4E). When all substitutions were combined (rMeV^SSPE-F^), infection resulted in smaller plaques than rMeV^WT^. Overall, this implies that N465I by itself results in enhanced cell-to-cell spread, which is reversed by all SSPE-F substitutions combined.

### rMeV^SSPE-F^ is strictly neurotropic

**Fig 5:**
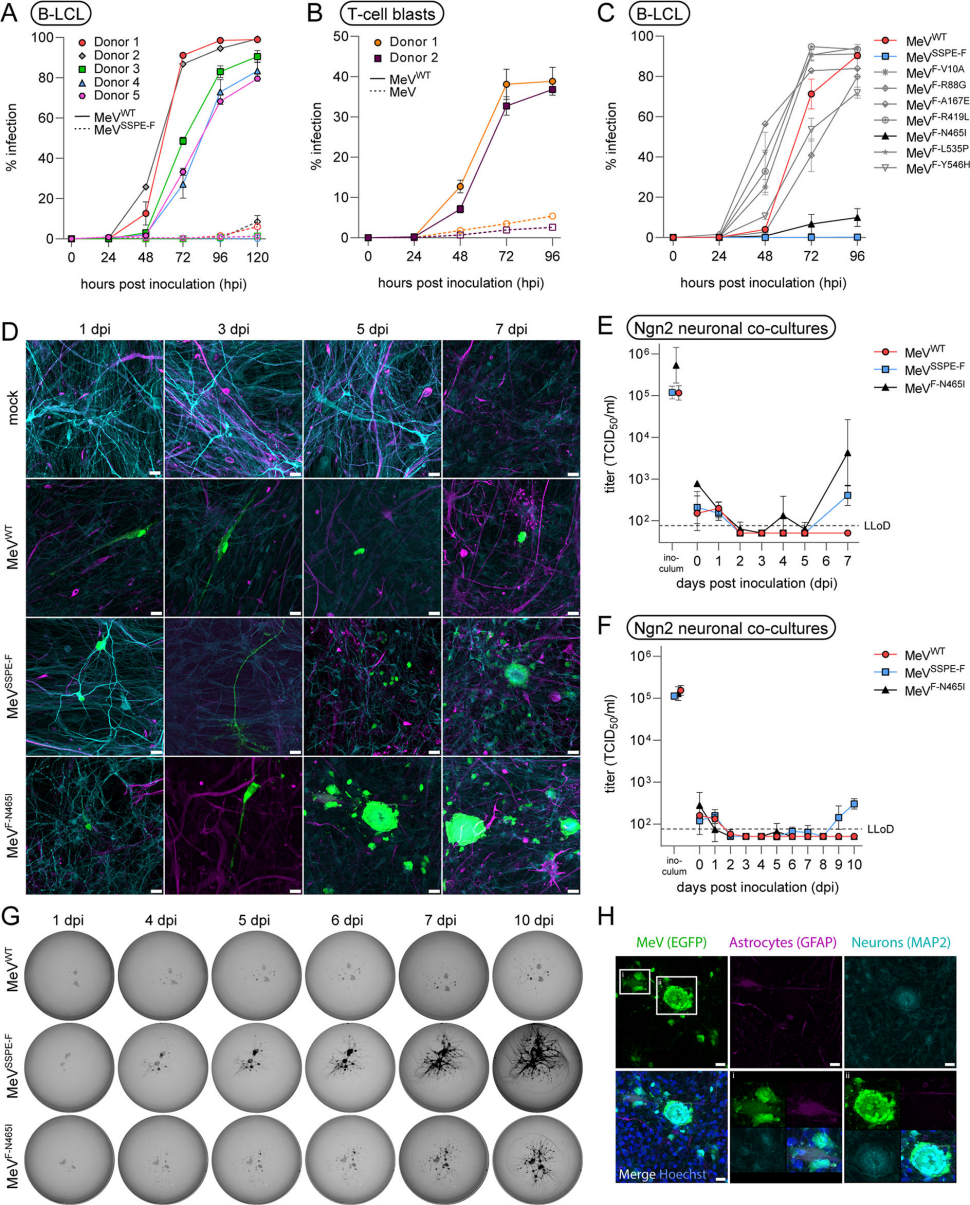
rMeV^SSPE-F^ replication in lymphoid cells and Ngn2 neuronal co-cultures. (**A–C**) Growth curve of rMeV^WT^ and rMeV^SSPE-F^ in B-LCL (**A**) and in T-cell blasts (**B**) and of single rMeV-F mutant viruses in B-LCL (**C**). Growth curves were performed in triplicate in one (**A and B**) or two (**C**) independent experiments, and error bars depict the SEM. (**D**) Visualization of rMeV^WT^, rMeV^SSPE-F^, and rMeV^F-N465I^ spread in Ngn2 neuronal co-cultures. Astrocytes are visualized in magenta, neurons in turquoise, and rMeV in green. Images were taken at indicated time points using a Zeiss LSM 700 confocal microscope at 10× magnification. Scale bars correspond to 20 µM. (**E and F**) Quantification of cell-free virus in inoculated Ngn2 neuronal co-cultures in two independent experiments: **E** represents the experiment shown in **D**; **F** represents the experiment shown in **G**. The geometric mean and SD are shown. (**G**) Visualization of rMeV^WT^-, rMeV^SSPE-F^-, and rMeV^F-N465I^-inoculated Ngn2 neuronal co-cultures cultured on cover slips in 24 wells. Gray shows clusters of neurons and astrocytes. Individual clusters are interconnected by neuronal extensions (not visible). Black visualizes the expression of EGFP (encoded by rMeV) and depicts the progressive spread over time. (**H**) Visualization of rMeV^F-N465I^-induced syncytia in Ngn2 neuronal co-cultures. Astrocytes are visualized in magenta, neurons in turquoise, MeV in green, and nuclei in blue. Images were taken using a Zeiss LSM 700 confocal microscope at 10× magnification. Scale bars correspond to 20 µM.

Since the patient SSPE virus efficiently disseminated throughout the brain, likely facilitated by its hyperfusogenic properties, we next evaluated the tropism of rMeV^SSPE-F^. First, we inoculated Epstein-Barr virus (EBV)-transformed B-lymphoblastic cell lines (B-LCLs) obtained from five different donors, a lymphoid target of MeV with high CD150 expression, with rMeV^WT^ and rMeV^SSPE-F^ (Fig. 5A). As expected, rMeV^WT^ productively replicated and disseminated in all B-LCLs as determined by the detection of enhanced green fluorescent protein (EGFP) by flow cytometry. However, we did not detect dissemination of rMeV^SSPE-F^ in B-LCL until 96 hpi and only limited replication in the subsequent 24 hours. Similar results were obtained in primary human T-cell blasts, in which rMeV^WT^ replicated efficiently and infected up to 40% of the cells at 96 hpi, but rMeV^SSPE-F^ did not exceed 6% infection at 96 hpi (Fig. 5B). To determine whether a specific mutation in the SSPE-F gene was responsible for this phenotype, we inoculated one B-LCL with the viruses harboring single amino acid substitutions in the F protein. Growth kinetics similar to rMeV^WT^ were observed for six of seven mutants (Fig. 5C). Infection percentages of different mutants varied at 48 and 72 hpi but reached between 70% and 100% by 96 hpi. In contrast, rMeV^F-N465I^ had an attenuated growth phenotype, resembling the phenotype observed for the complete rMeV^SSPE-F^.

Inoculation of human-induced pluripotent stem cell (hiPSC)-derived neurogenin-2 (Ngn2) neuronal co-cultures, consisting of neurons and astrocytes that both lack MeV receptors CD150 and nectin-4, led to productive infection and replication of both rMeV^SSPE-F^ and rMeV^F-N465I^ (Fig. 5D). rMeV^WT^ did not spread, and only single infected cells could be observed over the course of 7 days. During the experiment, we sampled supernatants of inoculated cultures daily to determine the amount of cell-free virus (Fig. 5E). No cell-free virus could be detected after infection with rMeV^WT^, and only a little cell-free virus could be detected after 7 dpi for rMeV^SSPE-F^ and rMeV^F-N465I^, suggesting that the spread of these viruses is primarily facilitated by cell-to-cell interactions. Upon repetition in new Ngn2 neuronal co-cultures over a time course of 10 days, similar results were obtained, although cell-free rMeV^SSPE-F^ was measured even later (9 dpi) and not at all for rMeV^F-N465I^, confirming primarily cell-to-cell spread (Fig. 5F). As Ngn2 neuronal co-cultures are sensitive to washing, the inoculum was not washed away and, therefore, still detected 1 dpi. Cell-to-cell spread, assessed by daily detection of EGFP, was faster for rMeV^SSPE-F^ when compared to rMeV^F-N465I^ (Fig. 5G). Upon phenotyping the cell tropism of rMeV, we observed a preference for microtubule-associated protein 2 (MAP2)-positive neurons over glial fibrillary acidic protein (GFAP)-positive astrocytes, especially at early time points. rMeV^SSPE-F^ and rMeV^F-N465I^ formed syncytia that included both astrocytes and neurons, as determined by co-staining of EGFP, GFAP, and MAP2, but single infected astrocytes or syncytia harboring only neurons could also be observed (Fig. 5H). Combined, we detected a tropism switch of rMeV^SSPE-F^ from lymphocytes to brain cells, in which the amino acid substitution N465I played an important role.

## DISCUSSION

Here, we describe the course of remdesivir treatment in a patient with Stage 3 fulminant SSPE. Transient clinical improvement was achieved before the patient ultimately succumbed to his infection. Post-mortem histopathological evaluation of the brain showed lymphoplasmacytic perivascular cuffs, loss of neurons, gliosis, white matter vacuolation, demyelination, and abundant presence of MeV RNA-positive cells but limited presence of MeV protein-expressing cells. The MeV sequence obtained revealed a typical SSPE virus genome with a hypermutated M gene and multiple mutations in the F gene including the cytoplasmic tail. Upon functional characterization, we detected a hyperfusogenic F protein, a phenotype facilitated by an amino acid substitution at position 465 (N465I). rMeV harboring this substitution, or all mutations of the F gene as detected in the patient’s brain, was strictly neurotropic and could no longer infect lymphocytes.

To assess the initial success of treatment, we relied on clinical observations. Evaluation of myoclonic seizures showed that the first two courses led to patient improvement, whereas the third course of remdesivir treatment proved ineffective. At this time, the patient developed gastrointestinal co-infections and pneumonia, leading to rapid deterioration. We did not detect mutations conferring remdesivir resistance, indicating that treatment failure was not due to the development of resistance. In contrast, the single amino acid substitution viruses and rMeV^SSPE-L^ appeared more sensitive to remdesivir, potentially because those viruses replicated slower compared to rMeV^WT^.

To date, no licensed treatment is available for SSPE, and other experimental treatments with ribavirin, IFNα, and inosine pranobex have only had limited success (40). Treatment with a combination of IFNα and ribavirin occasionally leads to transient steady or improved neurological symptoms, but prolonged stabilization was only reported for three cases (41–43). To properly evaluate the efficacy of remdesivir in treating SSPE, similar single or combination treatments should be considered in more patients. Moreover, efforts for an earlier treatment onset should be made to limit neurological damage and to maximize treatment success.

In a post-mortem histological evaluation, we only detected limited MeV-N protein-expressing cells in the brain but abundant N-RNA-positive cells. Interestingly, Miyahara et al. recently described two SSPE cases treated with antivirals and showed less MeV protein present in the brain when compared to untreated cases. They concluded that antiviral therapies may reduce the SSPE viral load in the CNS (44). The authors did not investigate the presence of viral RNA in these cases. An earlier study similarly reported the presence of viral RNA, but no or little viral protein, in cases experiencing long clinical disease (>36 months) (45). Future studies should assess if the RNA detected in the brain of SSPE patients is of genomic or mRNA origin.

Next, we characterized the F protein of the patient SSPE virus. The F protein plays a critical role in the clinical manifestation of SSPE and other MeV-related neurological complications (22–24, 26, 27, 34–39, 46–49). Multiple substitutions in the F protein have been described in literature, most of them contributing to a similar hyperfusogenic phenotype, consequently facilitating receptor-independent cell-to-cell spread. Some of the best-characterized substitutions are L454W (23, 27, 46, 47), T461I (35, 37, 47, 49), N462K/S (37, 47), and N465S/Y/K (37–39, 48). In this SSPE case, N465I was the substitution responsible for the hyperfusogenic phenotype. Previously, it was shown that the side chain length and molecular volume of the amino acid at position 465 influence fusion activity (39). A substitution with isoleucine led to enhanced fusogenicity when compared to asparagine (N), which was even more pronounced if the assay was performed at 30°C. The authors concluded that F proteins with an amino acid 465 of larger molecular volume have a lower energy barrier, facilitating conformational change of the F protein. In line, infection with rMeV^N465I^ resulted in significantly larger plaque sizes when compared to a recombinant WT-based virus. However, for rMeV^SSPE-F^, plaque sizes in Vero cells expressing the receptor were reduced, and the syncytium formation in receptor-negative Vero cells was relatively slow, indicating an additional influence of at least one additional amino acid substitution. It remains to be determined which factors influence the hyperfusogenic properties of this SSPE-F protein.

Functionally, the hyperfusogenic phenotype of the SSPE-F protein led to the efficient spread of rMeV^SSPE-F^ in Ngn2 neuronal co-cultures, which were used as a proxy for viral spread in the CNS. Similar experiments with comparable outcomes were previously performed for other hyperfusogenic viruses (25, 27). In NT2 neurons, the spread of the hyperfusogenic viruses was not accompanied by syncytium formation (25, 50), in contrast to our observations. Interestingly, we observed a complete tropism switch, as rMeV^SSPE-F^ could no longer spread in lymphocyte cultures expressing high levels of CD150. Seemingly, the SSPE virus had evolved to optimally replicate in cells of the brain and disseminate through the brain, losing its ability to spread in lymphocytes. This phenotype was also observed for rMeV^F-N465I^, corroborating the importance of this substitution.

The patient SSPE virus infected and disseminated efficiently in known receptor-negative human neural cells. While N465I was largely responsible for this phenotype, the exact mechanism of how rMeV^SSPE-F^ mediated known receptor-independent fusion in these cells remains to be elucidated. It has been suggested that the interaction of the H protein stalk with cell adhesion molecule (CADM)1 and CADM2, both expressed in the brain, facilitates the spread between neurons by triggering unstable F proteins (51–54). In addition, the spread of MeV genomes through pores between adhesive cells, transfer over synapses, and the nectin-1-orchestrated transfer of cytoplasmic cargo including infectious material was proposed (25, 50, 55). However, the latter process has only been shown for the transfer of cytoplasmic content of nectin-4-expressing epithelial cells as a mechanism to enter the CNS from infected respiratory epithelial cells, not between nectin-1-expressing neurons.

Altogether, this case encourages further investigation of remdesivir as a potential treatment of SSPE. Although remdesivir only led to transient clinical improvement in this patient, earlier remdesivir administration could further enhance treatment success by limiting viral replication in the CNS potentially leading to less neurological damage. The difficulty is that the incidence of SSPE is low and early diagnosis is difficult; therefore, evaluating the *in vivo* efficacy of drugs against SSPE in comparative studies will be challenging.

## MATERIALS AND METHODS

### Treatment and treatment evaluation

The patient received three courses (with a duration of 2 weeks each) of intravenous remdesivir. Dosages for the first course were 5 mg/kg/day on Day 1 and 2.5 mg/kg/day for the following 13 days. In the second and third course, the patient received 5 mg/kg/day for 14 days. Myoclonic seizures in hours per day were used as a quantitative proxy of clinical improvement.

### MeV genome detection

Total NAs were extracted directly from 200 µL of diagnostic CSF or brain tissue, using the MagNAPure 96 DNA and Viral NA Small Volume Kit (Roche Diagnostics) with 100 µL output eluate. Clinical materials were spiked with phocine distemper virus as an internal positive control for RNA virus detection. Extracted nucleic acids were tested by RT-PCR designed to detect MeV nucleoprotein.

### Illumina sequencing of MeV from post-mortem brain

Reverse transcription was performed on RNA extracted from the post-mortem brain using random hexamer primers and SuperScript III (Thermo Fisher Scientific); dsDNA synthesis was performed using Klenow (New England Biolabs). Libraries were prepared using the KAPA HyperPlus Kit (Roche Diagnostics) according to the manufacturer’s instructions with slight modifications. The shearing time was reduced to 3 min, and adapters were diluted 1:10. After the adapter ligation, an additional AMPure beads step was performed. Sequencing was performed on an Illumina MiSeq using the MiSeq Reagent KIT v3 (Illumina) to generate 2 × 300-bp reads. A coverage plot is shown in Fig. S4.

### Histopathology

The patient’s brain (parietal lobe, temporal lobe, occipital lobe, frontal lobe, basal ganglia, cerebellum, hippocampus, corpus callosum, brain stem, and periventricular regions), spinal cord, and lymphoid tissues (deep cervical lymph node and tonsils) were obtained post-mortem, fixed in 10% formaldehyde, and embedded in paraffin. The age-matched control brain was obtained post-mortem from a patient who succumbed with an intestinal malrotation. Tissues were cut sequentially at 5 µm and placed on Microscope KP FROST slides (Klinipath) for immunohistochemical evaluation or on Expredia SuperFrost Plus Adhesion slides (Thermo Fisher Scientific) for *in situ* hybridization. Hematoxylin and eosin staining and Kluver-Barrera staining were performed for histopathological evaluation and to evaluate the presence of myelin in different regions of the CNS. All slides used for comparison were stained in the same experiment.

To detect the MeV-N protein, slides were de-paraffinized and re-hydrated using 100% xylene and a decreasing ethanol series, followed by washing in phosphate-buffered saline (PBS). For antigen retrieval, slides were incubated in 0.1% protease solution for 10 min at 37°C. Tissues were blocked with 10% normal goat serum and incubated with primary mouse anti-MeV-N antibody (clone KK2, Chemicon) overnight at 4°C. After washing, slides were incubated with secondary antibody goat anti-mouse IgG1-biotin (SouthernBiotech) for 1 hour at room temperature (RT). Slides were washed, and streptavidin horseradish peroxidase (HRP; DAKO) was incubated for one additional hour at RT. HRP was revealed into a red precipitate with aminoethyl carbazole solution and counterstained in a 50% hematoxylin solution. Slides were mounted with Kaiser’s glycerol (Merck Millipore). Staining was imaged on a light microscope, and semi-quantitative analysis was performed. Scoring was performed from 0 to 3: 0, no positive cells were detected per high power field (hpf, magnification 10×); 1, ≤5 positive cells were detected per hpf; 2, between 5 and 20 positive cells were detected per hpf; and 3, ≥20 positive cells were detected per hpf. Ten hpfs were averaged per tissue.

MeV *in situ* hybridization was performed using a custom-designed probe targeting MeV-N RNA following the RNAscope 2.5 RED Kit (ACD Bio) instructions. Briefly, endogenous peroxidase was blocked, and antigen retrieval was performed by boiling slides in RNAscope target retrieval solution. Next, tissues were covered in RNAscope protease plus and incubated in a HybEz humidifying system. The target probe (anti-MeV nucleoprotein [NP]) followed by six amplification probes was incubated, and ultimately, the staining was developed using the fast red detection reagent in a 60:1 Fast RED A: Fast RED B ratio according to the manufacturer’s protocol. Slides were counterstained with a 50% hematoxylin solution (Gill no. 1) with subsequent bleaching in 0.02% ammonia water. Slides were mounted using EcoMount (Biocare Medical); imaged and semi-quantitative analysis was performed. Scoring was performed from 0 to 4: 0, no staining or <1 dot/10 cells were detected; 1, 1–3 dots/cell were detected; 2, 4–9 dots/cell and no or only a few clusters of dots were detected; 3, 10–15 dots/cell and/or <10% of dots in clusters were detected; and 4, >15 dots/cell and/or >10% of dots in clusters were detected.

### Cell culture

Human embryonic kidney 293T (HEK 293T), Vero, Vero cells expressing human SLAM (VHS) (56), and BSRT-7 cells were maintained in Dulbecco’s modified Eagle’s medium (DMEM) (Lonza) supplemented with 10% fetal bovine serum (FBS) (Sigma), 1,000-IU/mL penicillin, 100-µg/mL streptomycin, and 2-mM l-glutamine (Lonza) at 37°C in the presence of 5% CO_2_. Human EBV-transformed B-LCLs were cultured in Roswell Park Memorial Institute medium 1640 (RPMI-1640) (Lonza) supplemented with 10% FBS, 1,000-IU/mL penicillin, 100-µg/mL streptomycin, and 2-mM l-glutamine (R10F) at 37°C in 5% CO_2_. Primary T cells were obtained from peripheral blood mononuclear cells, isolated by gradient centrifugation using LymphoPrep (Stemcell), were washed four times in PBS, and were cultured in R10F in round bottom plates at a density of 0.3 × 10^6^ cells/well in the presence of 5-µg/mL concavalin A and 50-U/mL interleukin 2 (IL-2) for 4 days to generate T-cell blasts.

### Differentiation of hiPSCs to Ngn2 co-cultures

hiPSCs (WTC-11 Ngn2) were directly differentiated into excitatory cortical layer 2/3 neurons by doxycycline-induced overexpression of Ngn2 as described previously (57–59). At Day 0, hiPSCs were placed on Matrigel-coated (Corning, 10 µL in 1-mL KO DMEM, Life Technologies) coverslips. After 1 hour of cell attachment, wells were filled with Diff D0 medium [Stemflex medium (Thermo Fisher Scientific), 100-IU/mL penicillin, 100-µg/mL streptomycin, 1× fresh RevitaCell (Thermo Fisher Scientific), and 4-µg/mL doxycycline (DOX, Sigma Aldrich). On Day 1, the medium was refreshed with differentiation medium [Advanced DMEM/F12 (Thermo Fisher Scientific), 100-IU/mL penicillin, 100-µg/mL streptomycin, 0.1-mM non-essential amino acids (Lonza), 1% N2 supplement (Thermo Fisher Scientific), 10-ng/mL fresh human recombinant neurotrophin-3 (NT3, Stemcell Technologies), 10-ng/mL fresh brain-derived neurotrophic factor (BDNF, Prospectbio), 200-ng/mL fresh laminin (Corning), and 4-µg/mL DOX]. To guarantee the formation of functional synapses within the network, hiPSC-derived astrocytes were added to the culture in a 1:1 ratio (60). On Day 3, the medium was refreshed with Ngn2 medium [Neurobasal medium (Thermo Fisher Scientific), 100-IU/mL penicillin, 100-µg/mL streptomycin, 2-mM glutamine, 2% B27 minus RA supplement (Thermo Fisher Scientific), 10-ng/mL NT3, 10-ng/mL BDNF, and 4-µg/mL DOX]. Half of the medium was refreshed every other day during differentiation and maturation. After 21 days, the neural co-cultures were considered mature.

### Viruses

rMeV was engineered to express EGFP on position 1 of the genome, and all mutants were engineered to have one or multiple mutations in the F or L gene. rMeV was rescued in BSRT-7 cells by co-transfection of five plasmids encoding the MeV antigenome, MeV-N, MeV-P, MeV-L, and the T7 polymerase using Lipofectamine 3000. EGFP-expressing cells were overlayed on VHS cells to generate a p0 stock. Stocks were further grown and titrated on VHS cells. Single SSPE-F substitutions were cloned into a genotype B3 backbone to generate rMeV^F-V10A^, rMeV^F-R88G^, rMeV^F-A167E^, rMeV^F-R419L^, rMeV^F-N465I^, rMeV^F-L535P^, and rMeV^F-Y546H^. Additionally, rMeV^WT^, rMeV^SSPE-F^ (V10A R88G A167E R419L N465I L535P Y546H V457A; all major SSPE-F substitutions), and rMeV^SSPE-L^ (R623K A758T L920I R929K I1023T S1595N S1780P M1903I; all major SSPE-L substitutions) were generated in the B3 backbone. Single SSPE-L mutations were cloned into an IC323 background (rMeV^WT_IC323^, genotype D3) to generate rMeV^L-A758T^, rMeV^L-I1023T^, and rMeV^L-R1818G^.

### MeV-L protein modeling

The WT MeV-L protein was predicted using AlphaFold2 (https://cosmic-cryoem.org/tools/alphafold2/) (61), and individual domains and single amino acid substitutions were highlighted in University of California, San Francisco (UCSF) Chimera and PyMOL.

### Antiviral assay

Monolayers of VHS cells in 96-well plates were infected with rMeV^WT_IC323^, rMeV^L-A758T^, rMeV^L-I1023^, rMeV^L-R1818G^, rMeV^WT^, or rMeV^SSPE-L^ at multiplicity of infection (MOI) 0.05. Remdesivir was titrated in twofold dilutions starting at 50 µM. Control wells were incubated without remdesivir. After incubation at 37°C for 72 hours, cells were fixed with 4% paraformaldehyde (PFA), and plates were scanned using a Celigo Image Cytometer (Nexcelom) or a C.T.L. ImmunoSpot analyzer. The green fluorescent area was calculated using ImageJ and was expressed in percentage relative to the control wells.

### Fusion assay

The complementary fusion assay was performed as reported previously (26). Briefly, HEK-293T cells were transfected with MeV-F or MeV-F and MeV-H and the beta-galactosidase alpha-subunit expression plasmids in Opti-MEM (Thermo Scientific) using Lipofectamine 3000 (Invitrogen) to transiently express MeV glycoproteins. A second set of HEK-293T cells were transfected with MeV receptor expression plasmids (CD150, nectin 4, or no receptor) and the beta-galactosidase omega-subunit expression plasmid. Four hours after transfection, MeV glycoprotein-expressing cells were overlayed with MeV receptor-expressing cells. Cell-to-cell fusion led to beta-galactosidase complementation, which was stopped 20 hours after start of co-culture by cell lysis. The cell lysate was incubated with Galacton-Star substrate (Applied Biosystems), and luminescence was measured on an Infinite M1000Pro microplate reader (Tecan).

### Plaque assay

To evaluate the effect of single SSPE-F substitutions, 12-well plates of VHS were inoculated in triplicate with 100 TCID_50_ of each rMeV harboring a single SSPE-F substitution. rMeV^F-L454W^ (23), previously shown to harbor a hyperfusogenic F protein, was used as an assay control. After adsorption for 1 hour at 37°C, cells were washed, an overlay of 1.6% Avicell in OptiMEM was added, and plates were incubated for 3 days at 37°C. The overlay was removed, plates were washed with PBS and fixed with 4% PFA, and EGFP expression was visualized on an Amersham Typhoon. Plaque size was evaluated in ImageQuant and presented as percentage of the average plaque size calculated for rMeV^WT^.

### Infection of lymphocyte cultures

B-LCL and primary T-cell blasts were pelleted and infected with rMeV^WT^ and rMeV^SSPE-F^ at MOI 0.01 for 1 hour at 32°C or at MOI 0.1 for 1 hour at 37°C, respectively. After incubation, cells were washed with R10F and incubated at 37°C for the indicated time points. The infection percentage was determined by flow cytometry by measuring the expression of EGFP and analyzed using FlowJo v10.8.1.

### Infection of Ngn2 co-cultures

hiPSC-derived Ngn2 co-cultures were infected with 20,000 viral particles of rMeV^WT^, rMeV^SSPE F^, or rMeV^F-N465I^ in Ngn2 medium for 1 hour at 37°C. Without washing, the medium was replaced with fresh Ngn2 medium, and cultures were incubated at 37°C for 7–10 days. The supernatant was sampled daily, and end-point titrations were performed on VHS cells in threefold dilution steps. Plates were imaged daily on an Amersham Typhoon to follow the progressive spread of EGFP within Ngn2 cultures as a proxy for MeV replication. For immunofluorescent labeling, cells were fixed after 7–10 days using 10% formalin, permeabilized with 1% Triton X-100, and blocked with 0.5% Triton X-100 and 1% bovine serum albumin (Sigma). Slides were incubated with primary guinea pig anti-MAP-2 (1:200; Synaptic Systems) and rabbit anti-GFAP (1:200, Millipore) in a blocking buffer for 1 hour at RT. Secondary antibody incubation was performed for 1 hour at RT using donkey anti-guinea pig/Alexa-647 (1:500) and donkey anti-rabbit Alexa-555 (1:500) (Jackson ImmunoResearch) in a blocking buffer. Hoechst (Invitrogen) was used to visualize nuclei, and slides were mounted in ProLong Antifade Mountant (Thermo Fisher) and imaged using a Zeiss LSM 700 confocal microscope.

### Statistical analysis

Statistical analysis was performed in GraphPad Prism 9.5.0. Data were evaluated for normal distribution, and Bonferroni correction was performed where applicable. Statistical tests and *P*-values are indicated per experiment in the figure legends.

## Data Availability

The obtained genome sequence was deposited in GenBank with the accession number ON024067.

## SUPPLEMENTAL MATERIAL

Supplemental material jvi.01874-23-s0001.docx Supplemental methods, Figures S1 to S4, and Tables S1 and S2.

ASM does not own the copyrights to Supplemental Material that may be linked to, or accessed through, an article. The authors have granted ASM a non-exclusive, world-wide license to publish the Supplemental Material files. Please contact the corresponding author directly for reuse.
