# Supplementary material for: Functional properties of measles virus proteins derived from a subacute sclerosing panencephalitis patient who received repeated remdesivir treatments
Source: J Virol. 2024 Feb 8;98(3):e01874-23. doi: 10.1128/jvi.01874-23 (PMC10949486; doi:10.1128/jvi.01874-23)
Supplement: Supplemental material — Supplemental methods, Figures S1 to S4, and Tables S1 and S2. [file jvi.01874-23-s0001.docx]

*Supplementary material belonging to*

**Functional properties of measles virus proteins derived from a subacute sclerosing panencephalitis patient who received repeated remdesivir treatments**

Katharina S. Schmitz^1^*, Kim Handrejk^1^*, Lelde Liepina^2^, Lisa Bauer^1^, Griffin D. Haas^3^, Fabiënne van Puijfelik^1$^, Edwin J.B. Veldhuis Kroeze^1^, Marta Riekstina^4^, Jurgis Strautmanis^2^, Huyen Cao^5^, Rob M. Verdijk^6^, Corine H. GeurtsvanKessel^1^, Sander van Boheemen^1^, Debby van Riel^1^, Benhur Lee^3^, Matteo Porotto^7,8,9^, Rik L. de Swart^1^, and Rory D. de Vries^1#^

^1^ Department of Viroscience, Erasmus MC, Rotterdam, the Netherlands

^2^ Clinic for Pediatric Neurology and Neurosurgery, Children's Clinical University Hospital, Riga, Latvia

^3^ Department of Microbiology, Icahn School of Medicine at Mount Sinai, New York, NY, USA

^4^ Department of Pathology, Children's Clinical University Hospital, Riga, Latvia

^5^ Departments of Clinical Research, Biometrics, and Virology, Gilead Sciences, Inc., Foster City, CA, USA

^6^ Department of Pathology, Erasmus MC University Medical Center Rotterdam, Rotterdam, the Netherlands

^7^ Department of Pediatrics, Columbia University Irving Medical Center, New York, NY, USA.

^8^ Center for Host–Pathogen Interaction, Columbia University Irving Medical Center, New York, NY, USA.

^9^ Department of Experimental Medicine, University of Campania “Luigi Vanvitelli,” Caserta, Italy

^$^ Current affiliation: Department of Immunology, Erasmus MC, Rotterdam, the Netherlands

* Authors contributed equally

^#^ Correspondence: Rory D. de Vries ([r.d.devries@erasmusmc.nl](mailto:r.d.devries@erasmusmc.nl), +31 10 704 4099)

**Keywords:** measles, subacute sclerosing panencephalitis, remdesivir, fusion, resistance

**Additional methods**

**Serology.** Levels of measles virus-specific IgG were measured by performing an in-house–developed ELISA as described previously (1). Briefly, ELISA plates were coated with BPL-inactivated whole measles virus at 4°C overnight. After coating, plates were blocked, washed, and incubated with a 3-fold dilution series of plasma (1:30 to 1:5,314,410) at 37°C for 2 hours. Plates were washed and horseradish peroxidase–labelled rabbit anti-human IgG was added. Plates were incubated at 37°C for 1 hour, washed, and developed by using 3,3′,5,5′-tetramethylbenzidine (KPL). Plates were measured at an OD of 450 nm (OD_450_) using an ELISA microtiter plate reader (Tecan). OD450 signal was corrected by subtracting background signal in the OD620 channel. Human pooled plasma (HPP) and intravenous immunoglobulins (IVIg) were used as positive controls.


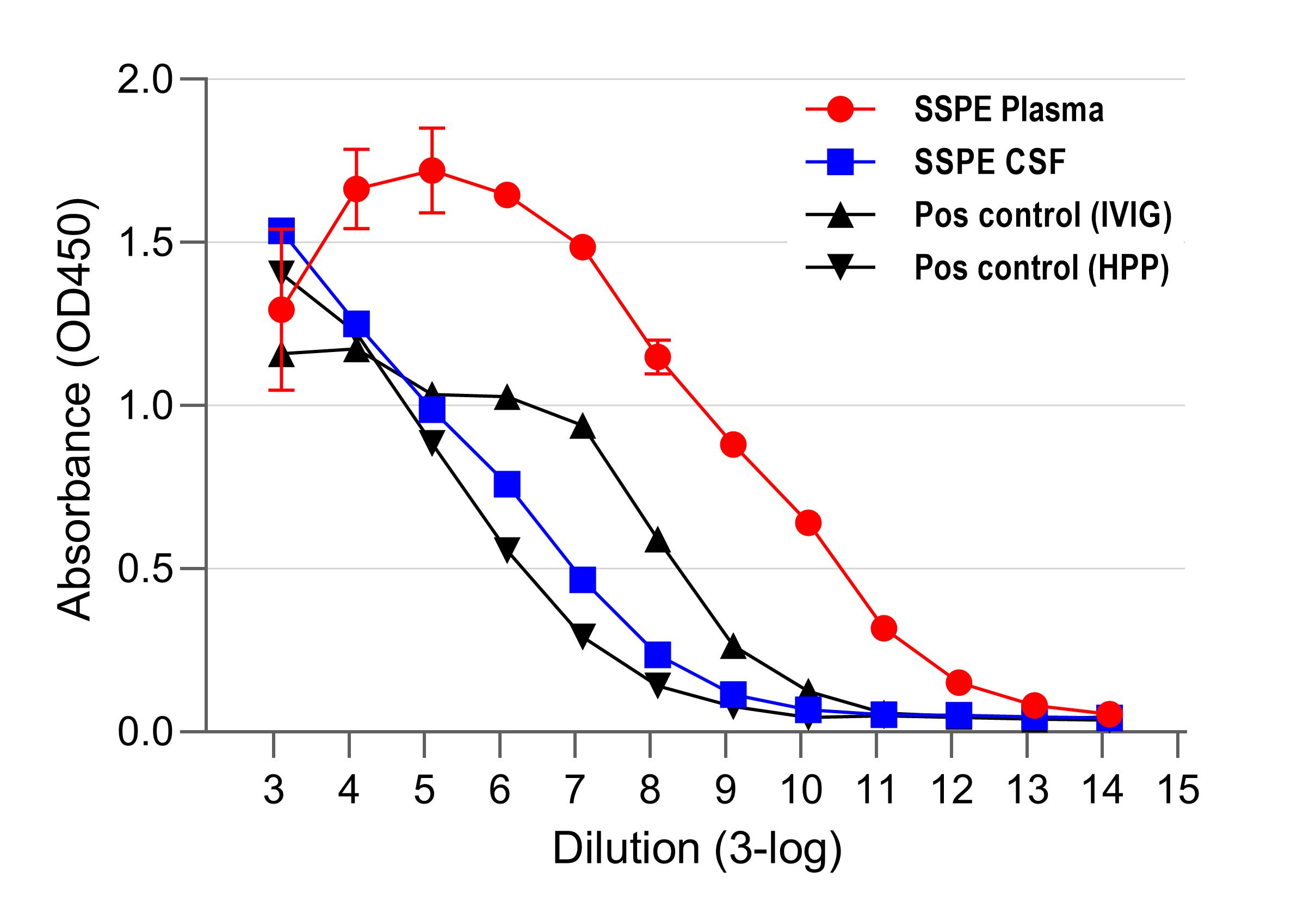


**Supplementary Figure 1. BPL-MeV ELISA.** Plasma and CSF of SSPE patient, IVIG (intravenous immunoglobulins) and human pooled plasma (HPP) were 3-fold serially diluted and used in ELISA using β-propiolactone-inactivated MeV as antigen.


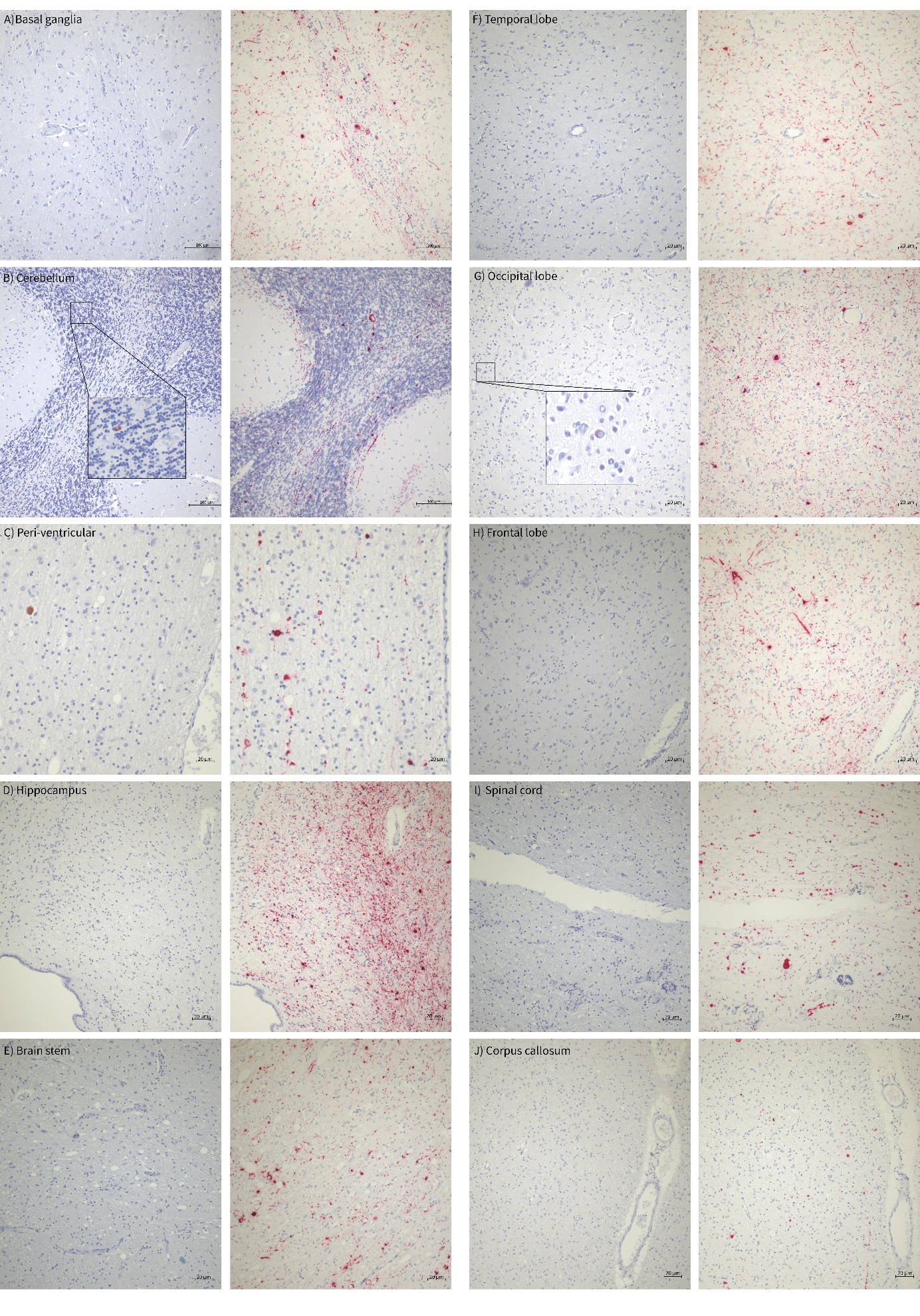


**Supplementary Figure *2*. Nucleoprotein detection in patient brain.** IHC (protein detection, left) and ISH (RNA detection, right) in consecutive slides of indicated brain parts. A semi-quantitative evaluation is shown in Supplementary table 1.

**
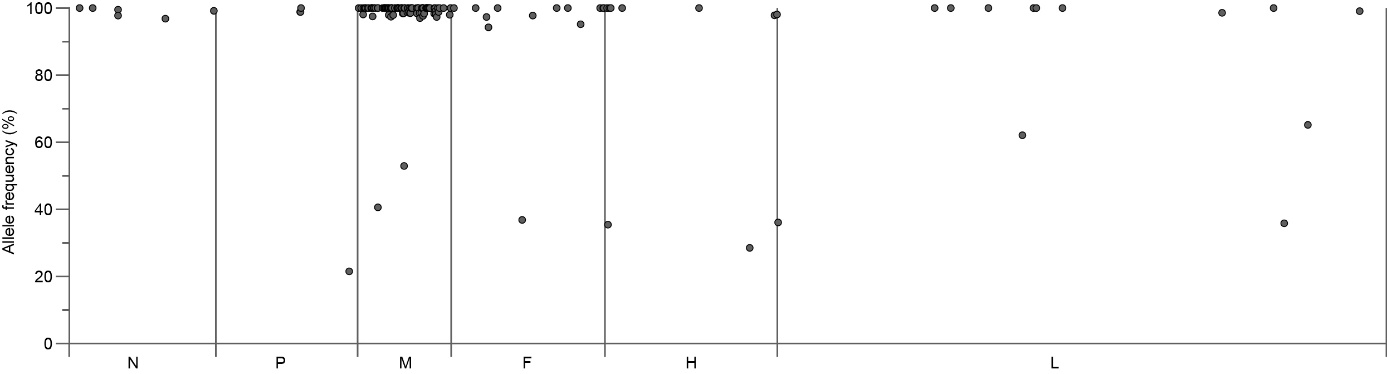
**

**Supplementary Figure 3.** SSPE virus genome nucleotide changes in comparison to the closest wildtype measles virus genome sequence (accession number: KY969477.1). Every dot represents a mutation detected in the indicated percentage of sequencing reads. Only the protein-coding sequence is displayed.


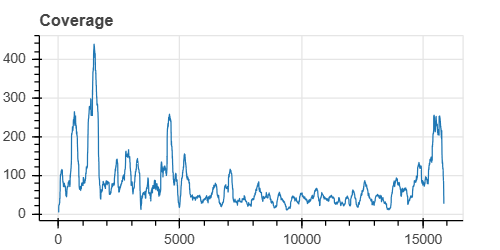


**Supplementary Figure 4.** Coverage plot of illumina sequencing of MeV from post-mortem brain. The y-axis is the read number and the x-axis the genome position. The total number of reads was 15,677,750 of which 0.06% reads mapped to MeV.

**Supplementary Table 1:** Scoring of IHC (protein detection) and ISH (RNA detection) staining of tissue from SSPE patient. Per tissue 10 high power fields (hpf, magnification: 10x) were averaged. Corresponds to Supplementary Figure 2.

| **Tissue** | **IHC score** | **ISH score** |
| --- | --- | --- |
| Cerebellum | 1 | 3 |
| Parietal lobe | 1 | 4 |
| Basal ganglia | 3 | 3 |
| Peri-ventricular | 1 | 3 |
| Temporal lobe | 2 | 2 |
| Occipital lobe | 2 | 3 |
| Frontal lobe | 1 | 2 |
| Hippocampus | 1 | 4 |
| Corpus callosum | 0 | 1 |
| Brain stem | 2 | 3 |
| Spinal cord | 2 | 2 |
| Deep cervical lymph node | 0 | 0 |
| Tonsils | 0 | 0 |

| **IHC scoring** | |
| --- | --- |
| **0** | No positive cells/hpf |
| **1** | ≤ 5 positive cells/hpf |
| **2** | ≥ 5 ≤ 20 positive cells/hpf |
| **3** | ≥ 20 positive cells/hpf |
|  |  |
| **ISH scoring** | |
| **0** | No staining or < 1 dot/10 cells |
| **1** | 1-3 dots/cell |
| **2** | 4-9 dots/cell and no or few clusters of dots |
| **3** | 10-15 dots/cell and/or <10% of dots in clusters |
| **4** | >15 dots/cell and/or >10% of dots in clusters |

**Supplementary Table 2.** SSPE virus genome amino acid and nucleotide changes in comparison to the closest wildtype measles virus genome (accession number: KY969477.1). Corresponds to Supplementary Figure 3.

| **Protein** | **Amino acid change** | **Nucleotide change** | **Allele frequency (%)** |
| --- | --- | --- | --- |
| **N** | P38S | C112T | 100.00 |
|  | G85S | G253A | 100.00 |
|  | V176I | G526A | 99.55 |
|  | V176A | T527C | 97.75 |
|  | N345K | C1035A | 96.91 |
|  | N519N | T1557C | 99.24 |
| **P** | G303G | A909G | 98.88 |
|  | G304E | G911A | 98.86 |
|  | Y306H | T916C | 100.00 |
|  | R479G | C1435G | 21.54 |
| **M** | Y5H | T13C | 100.00 |
|  | I14T | T41C | 100.00 |
|  | I21T | T62C | 98.11 |
|  | I21I | T63C | 100.00 |
|  | Y26H | T76C | 100.00 |
|  | S27S | T81C | 100.00 |
|  | L31P | T92C | 100.00 |
|  | V37A | T110C | 100.00 |
|  | P40P | T120C | 100.00 |
|  | F50L | T148C | 100.00 |
|  | F50F | T150C | 100.00 |
|  | Y52H | T154C | 100.00 |
|  | M53T | T158C | 100.00 |
|  | F54S | T161C | 100.00 |
|  | L55P | T164C | 97.5 |
|  | V59A | T176C | 100.00 |
|  | V59V | T177C | 100.00 |
|  | L65L | T193C | 100.00 |
|  | F73L | T217C | 100.00 |
|  | F73F | T219C | 100.00 |
|  | G74E | G221A | 40.62 |
|  | L91P | T272C | 100.00 |
|  | T95T | T285C | 100.00 |
|  | L97P | T290C | 100.00 |
|  | L97L | T291C | 100.00 |
|  | I99T | T296C | 100.00 |
|  | V101A | T302C | 100.00 |
|  | V101V | T303C | 100.00 |
|  | R103R | T309C | 100.00 |
|  | L107P | T320C | 100.00 |
|  | N108N | T324C | 100.00 |
|  | L111P | T332C | 100.00 |
|  | V112A | T335C | 100.00 |
|  | F113L | T337C | 97.92 |
|  | Y114H | T340C | 100.00 |
|  | L119P | T356C | 100.00 |
| **M** | T120T | T360C | 100.00 |
|  | L121P | T362C | 97.44 |
|  | L122P | T365C | 100.00 |
|  | P124P | T372C | 100.00 |
|  | W125R | T373C | 100.00 |
|  | L129P | T386C | 97.96 |
|  | F135L | T403C | 100.00 |
|  | F135S | T404C | 100.00 |
|  | N142N | T426C | 100.00 |
|  | V144A | T431C | 100.00 |
|  | V144V | T432C | 100.00 |
|  | N145N | T435C | 100.00 |
|  | L146P | T437C | 100.00 |
|  | I147T | T440C | 100.00 |
|  | D150D | T450C | 100.00 |
|  | R156R | T486C | 98.44 |
|  | V157A | T470C | 100.00 |
|  | V158V | T474C | 100.00 |
|  | Y159H | T475C | 100.00 |
|  | M160T | T479C | 100.00 |
|  | L165L | T495C | 98.48 |
|  | D167D | T501C | 100.00 |
|  | D167G | A500G | 52.94 |
|  | Y170H | T508C | 100.00 |
|  | Y170Y | T510C | 100.00 |
|  | F180L | T538C | 98.73 |
|  | F180S | T539C | 100.00 |
|  | S182P | T544C | 100.00 |
|  | N184N | T552C | 98.84 |
|  | F188S | T563C | 98.53 |
|  | F188L | T562C | 100.00 |
|  | L190P | T569C | 98.53 |
|  | V192A | T575C | 100.00 |
|  | L194P | T581C | 100.00 |
|  | L194L | T582C | 100.00 |
|  | I196T | T587C | 100.00 |
|  | L212P | T635 | 100.00 |
|  | P213P | T639C | 98.48 |
|  | F217S | T650C | 100.00 |
|  | F217F | T651C | 100.00 |
|  | M218T | T653C | 100.00 |
|  | F224L | T670C | 98.53 |
|  | F224S | T671C | 97.06 |
|  | S229S | T687C | 98.51 |
|  | Y232H | T694C | 100.00 |
|  | S233S | T699C | 100.00 |
|  | S233P | T697C | 100.00 |
|  | D235D | T705C | 100.00 |
|  | Y236H | T706C | 97.73 |
|  | Y236Y | T708C | 100.00 |
| **M** | C237R | T709C | 100.00 |
|  | M239T | T716C | 98.41 |
|  | L246P | T737C | 100.00 |
|  | V247V | T741C | 100.00 |
|  | F248L | T742C | 100.00 |
|  | F248F | T744C | 100.00 |
|  | F248S | T743C | 100.00 |
|  | L250P | T749C | 100.00 |
|  | L250L | T750C | 100.00 |
|  | I253T | T758C | 100.00 |
|  | G254G | A762G | 100.00 |
|  | L258L | T774C | 100.00 |
|  | L258P | T773C | 100.00 |
|  | I260T | T779C | 100.00 |
|  | F276L | T826C | 98.36 |
|  | F276S | T827C | 100.00 |
|  | L280L | T838C | 100.00 |
|  | C281R | T841C | 99.1 |
|  | C281C | T843C | 98.21 |
|  | L284P | T851C | 97.41 |
|  | N288N | T864C | 100.00 |
|  | L291P | T872C | 98.92 |
|  | L291L | T873C | 98.91 |
|  | L294S | T881C | 100.00 |
|  | L309P | T926C | 100.00 |
|  | L331P | T992C | 98.08 |
|  | V334A | T100.001C | 100.00 |
| **F** | V10A | T29C | 100.00 |
|  | R88G | A262G | 100.00 |
|  | T127T | A381G | 97.37 |
|  | G134G | C402T | 94.29 |
|  | A167E | C500A | 100.00 |
|  | D255N | G763A | 36.84 |
|  | G293G | G879A | 97.78 |
|  | R378R | A1134G | 100.00 |
|  | R419L | G1256T | 100.00 |
|  | N465I | A1394T | 95.24 |
|  | L535P | T1604C | 100.00 |
|  | Y546H | T1636C | 100.00 |
|  | V547A | T1640C | 100.00 |
| **H** | I8T | T23C | 100.00 |
|  | F11S | T32C | 35.48 |
|  | Y17H | T49C | 100.00 |
|  | S21S | T63C | 100.00 |
|  | R62Q | G185A | 100.00 |
|  | P338L | C1013T | 100.00 |
|  | Q520R | A1559G | 28.57 |
|  | A608A | T1824C | 97.87 |
|  | X618Q | T1852C | 98.11 |
| **L** | S3S | G9A | 36.11 |
|  | I564I | C1692T | 100.00 |
|  | R623K | G1868A | 100.00 |
|  | A758T | G2272A | 100.00 |
|  | I879I | C2637T | 62.16 |
|  | L920I | C2758A | 100.00 |
|  | R929K | G2786A | 100.00 |
|  | I1023T | T3068C | 100.00 |
|  | S1595N | G4784A | 98.63 |
|  | S1780P | T5338C | 100.00 |
|  | R1818G | A5452G | 35.9 |
|  | M1903I | G5709T | 65.18 |
|  | V2088V | G6264A | 99.12 |

**References**

1. de Swart RL, de Vries RD, Rennick LJ, van Amerongen G, McQuaid S, Verburgh RJ, Yüksel S, de Jong A, Lemon K, Nguyen DT, Ludlow M, Osterhaus A, Duprex WP. 2017. Needle-free delivery of measles virus vaccine to the lower respiratory tract of non-human primates elicits optimal immunity and protection. NPJ Vaccines 2:22.
